# 4-(2-Hydroxy­benzyl­idene)-3-methyl­isoxazol-5(4*H*)-one

**DOI:** 10.1107/S1600536809045486

**Published:** 2009-11-07

**Authors:** Qingfang Cheng, Xing-you Xu, Li-sha Liu, Li Zhang

**Affiliations:** a Huaihai Institute of Technology, Lianyungang 222005, People’s Republic of China

## Abstract

The mol­ecular skeleton of the title mol­ecule, C_11_H_9_NO_3_, is approximately planar (r.m.s. deviation = 0.0056 Å); the two rings form a dihedral angle of 6.5 (1)°. In the crystal structure, inter­molecular O—H⋯N hydrogen bonds involving the H atom of the hydr­oxy group and the N atom of the isoxazole ring link mol­ecules into chains running along the *c* axis.

## Related literature

For the biological activity of aryl­methyl­ene isoxazolone derivatives, see: Ishioka *et al.* (2002 ▶); Liu *et al.* (2005 ▶). For related structures, see: Cocivera *et al.* (1976 ▶); Villemin *et al.* (1993 ▶); Zhang *et al.* (2008 ▶).
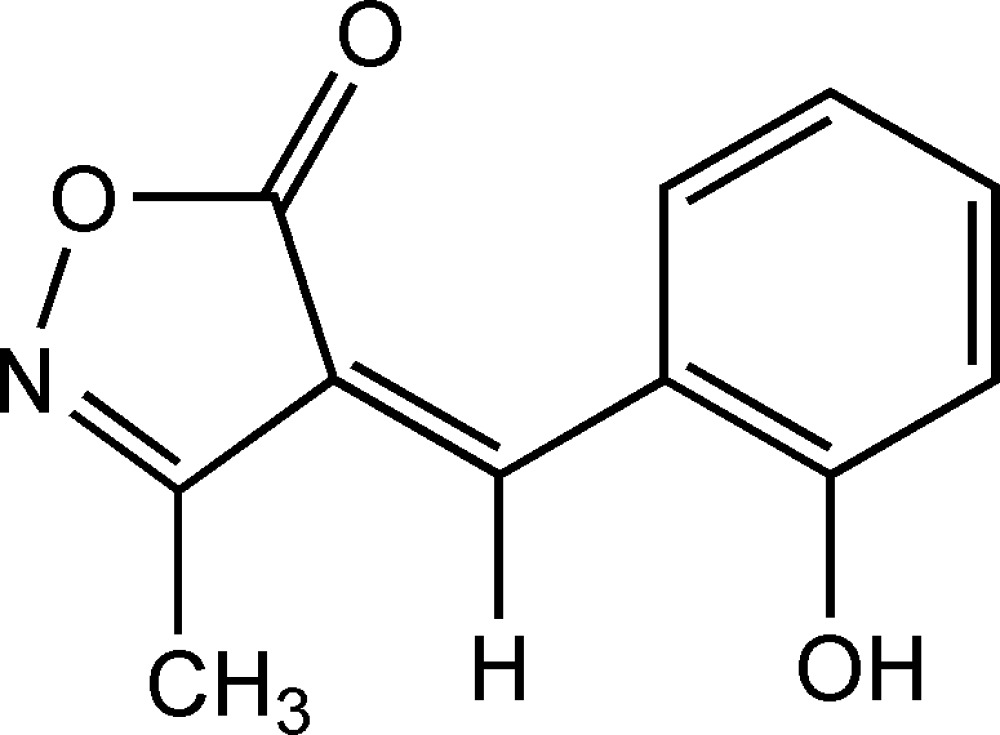


## Experimental

### 

#### Crystal data

C_11_H_9_NO_3_
*M* *_r_* = 203.19
Monoclinic, 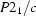

*a* = 8.0172 (12) Å
*b* = 6.8620 (9) Å
*c* = 17.535 (2) Å
β = 99.962 (2)°
*V* = 950.1 (2) Å^3^
*Z* = 4
Mo *K*α radiation
μ = 0.11 mm^−1^
*T* = 298 K
0.43 × 0.30 × 0.28 mm

#### Data collection

Bruker SMART CCD area-detector diffractometer
Absorption correction: multi-scan (*SADABS*; Sheldrick, 1996 ▶) *T*_min_ = 0.956, *T*_max_ = 0.971
4598 measured reflections
1669 independent reflections
1067 reflections with *I* > 2σ(*I*)
*R*_int_ = 0.034

#### Refinement

*R*[*F* ^2^ > 2σ(*F* ^2^)] = 0.043
*wR*(*F* ^2^) = 0.125
*S* = 1.03
1669 reflections
137 parameters
H-atom parameters constrained
Δρ_max_ = 0.33 e Å^−3^
Δρ_min_ = −0.18 e Å^−3^

### 

Data collection: *SMART* (Bruker, 2000 ▶); cell refinement: *SAINT* (Bruker, 2000 ▶); data reduction: *SAINT*; program(s) used to solve structure: *SHELXS97* (Sheldrick, 2008 ▶); program(s) used to refine structure: *SHELXL97* (Sheldrick, 2008 ▶); molecular graphics: *SHELXTL* (Sheldrick, 2008 ▶) and *DIAMOND* (Brandenburg, 2004 ▶); software used to prepare material for publication: *SHELXTL*.

## Supplementary Material

Crystal structure: contains datablocks I, global. DOI: 10.1107/S1600536809045486/cv2639sup1.cif

Structure factors: contains datablocks I. DOI: 10.1107/S1600536809045486/cv2639Isup2.hkl

Additional supplementary materials:  crystallographic information; 3D view; checkCIF report

## Figures and Tables

**Table 1:** Hydrogen-bond geometry (Å, °)

*D*—H⋯*A*	*D*—H	H⋯*A*	*D*⋯*A*	*D*—H⋯*A*
O3—H3⋯N1^i^	0.82	2.07	2.852 (2)	159

Symmetry code: (i) 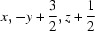.

**Fig. 1.**
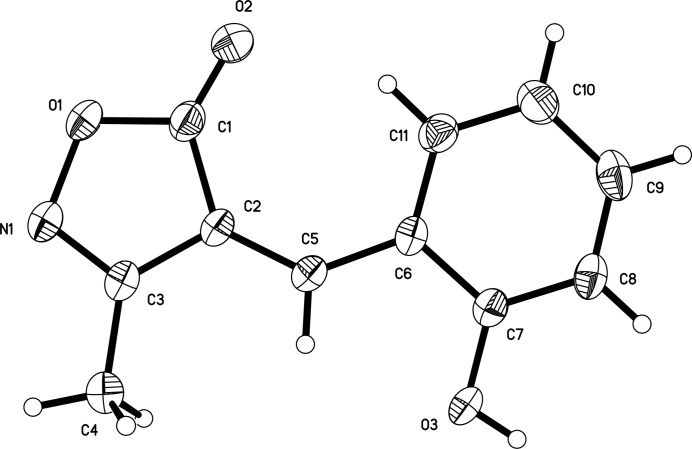
The molecular structure of the title complex. Displacement ellipsoids are drawn at the 30% probability level.

## supplementary crystallographic information

### Comment

Arylmethylene isoxazolone derivatives are effective anti-psychotics in the treatment of depression and schizophrenia. The study about arylmethylene isoxazolone derivatives mainly concentrates in the biological activities (Ishioka *et al.*, 2002; Liu *et al.*, 2005). However, structural studies of them have rarely been reported. As a part of our investigation on arylmethylene isoxazolone derivatives, we report here the structure of the title compound, (I), synthesized by three component condensation reaction of methyl acetoacetate, hydroxylamine with salicylaldehyde in aqueous media under ultrasonic irradiation.

In (I) (Fig. 1), all bond lengths and angles agree well with those reported for the related compounds (Cocivera *et al.*, 1976; Villemin *et al.*, 1993; Zhang *et al.*, 2008). The molecular structure adopts a *Z*-configuration about the C2=C5 double bond. The hydroxy groups and the isoxazol groups are involved in intermolecular O—H···N hydrogen bonds (Table 1), which link the molecules into chain structure, hydroxy O atom in the molecule acts as hydrogen-bond donor to isoxazol N atom in the neighbouring molecule, so forming a chain structure.

### Experimental

A mixture of methyl acetoacetate (4 mol), hydroxylamine hydrochloride (4 mmol), and pyridine(4 mmol) in distilled water(10 ml) was irradiated in the water bath of an ultrasonic cleaner for 10 min., then salicylaldehyde(4 mmol) was slowly added to the mixture. The resulting mixture was irradiated in the water bath of an ultrasonic cleaner for 1.5 h. The solution was left at room temperature overnight, the obtained mushy solution was filtered and the solid was washed with cold water and ethanol. The crude product was recrystallized from ethanol to afford the desired product as a yellow solid. Single crystal of (I) were obtained by slow evaporation of aqueous ethanol(95%) solution at ambient temperature after 7 d. Elemental analysis, calculated for C11 H9 N O3: C 65.02, H 4.46, N 6.89%; found: C 65.09. H 4.49, N 6.92%.

### Refinement

All hydrogen atoms were geometrically positioned (C—H = 0.93–0.96 A °, O—H = 0.82 A °) and allowed to ride on their parent atoms, with *U*_iso_(H) = 1.2-1.5*U*_eq_(C,*O*).

### Crystal data

**Table:**

C_11_H_9_NO_3_	*F*(000) = 424
*M**_r_* = 203.19	*D*_x_ = 1.420 Mg m^−^^3^
Monoclinic, *P*2_1_/*c*	Mo *K*α radiation, λ = 0.71073 Å
Hall symbol: -P 2ybc	Cell parameters from 1182 reflections
*a* = 8.0172 (12) Å	θ = 2.4–22.5°
*b* = 6.8620 (9) Å	µ = 0.11 mm^−^^1^
*c* = 17.535 (2) Å	*T* = 298 K
β = 99.962 (2)°	Prism, yellow
*V* = 950.1 (2) Å^3^	0.43 × 0.30 × 0.28 mm
*Z* = 4	

### Data collection

**Table:**

Bruker SMART CCD area-detector diffractometer	1669 independent reflections
Radiation source: fine-focus sealed tube	1067 reflections with *I* > 2σ(*I*)
graphite	*R*_int_ = 0.034
φ and ω scans	θ_max_ = 25.0°, θ_min_ = 2.4°
Absorption correction: multi-scan (*SADABS*; Sheldrick, 1996)	*h* = −9→9
*T*_min_ = 0.956, *T*_max_ = 0.971	*k* = −8→7
4598 measured reflections	*l* = −19→20

### Refinement

**Table:**

Refinement on *F*^2^	Primary atom site location: structure-invariant direct methods
Least-squares matrix: full	Secondary atom site location: difference Fourier map
*R*[*F*^2^ > 2σ(*F*^2^)] = 0.043	Hydrogen site location: inferred from neighbouring sites
*wR*(*F*^2^) = 0.125	H-atom parameters constrained
*S* = 1.03	*w* = 1/[σ^2^(*F*_o_^2^) + (0.0547*P*)^2^ + 0.2598*P*] where *P* = (*F*_o_^2^ + 2*F*_c_^2^)/3
1669 reflections	(Δ/σ)_max_ < 0.001
137 parameters	Δρ_max_ = 0.33 e Å^−^^3^
0 restraints	Δρ_min_ = −0.18 e Å^−^^3^

### Special details

**Table:**

Geometry. All e.s.d.'s (except the e.s.d. in the dihedral angle between two l.s. planes) are estimated using the full covariance matrix. The cell e.s.d.'s are taken into account individually in the estimation of e.s.d.'s in distances, angles and torsion angles; correlations between e.s.d.'s in cell parameters are only used when they are defined by crystal symmetry. An approximate (isotropic) treatment of cell e.s.d.'s is used for estimating e.s.d.'s involving l.s. planes.
Refinement. Refinement of *F*^2^ against ALL reflections. The weighted *R*-factor *wR* and goodness of fit *S* are based on *F*^2^, conventional *R*-factors *R* are based on *F*, with *F* set to zero for negative *F*^2^. The threshold expression of *F*^2^ > σ(*F*^2^) is used only for calculating *R*-factors(gt) *etc*. and is not relevant to the choice of reflections for refinement. *R*-factors based on *F*^2^ are statistically about twice as large as those based on *F*, and *R*- factors based on ALL data will be even larger.

### Fractional atomic coordinates and isotropic or equivalent isotropic displacement parameters (Å^2^)

**Table:**

	*x*	*y*	*z*	*U*_iso_*/*U*_eq_	
N1	0.1424 (3)	0.7335 (3)	0.29966 (10)	0.0495 (6)	
O1	0.2203 (2)	0.5472 (2)	0.29343 (8)	0.0573 (5)	
O2	0.3282 (3)	0.3078 (3)	0.37132 (10)	0.0668 (6)	
O3	0.1516 (2)	0.6467 (2)	0.64469 (8)	0.0579 (5)	
H3	0.1416	0.6505	0.6904	0.087*	
C1	0.2599 (3)	0.4642 (4)	0.36509 (12)	0.0474 (6)	
C2	0.2013 (3)	0.5995 (3)	0.41967 (12)	0.0372 (6)	
C3	0.1320 (3)	0.7584 (3)	0.37120 (12)	0.0404 (6)	
C4	0.0543 (3)	0.9391 (3)	0.39499 (13)	0.0541 (7)	
H4A	0.0185	1.0195	0.3504	0.081*	
H4B	0.1358	1.0085	0.4316	0.081*	
H4C	−0.0418	0.9065	0.4184	0.081*	
C5	0.2050 (3)	0.5923 (3)	0.49738 (12)	0.0388 (6)	
H5	0.1541	0.6992	0.5165	0.047*	
C6	0.2713 (3)	0.4536 (3)	0.55603 (11)	0.0377 (5)	
C7	0.2432 (3)	0.4893 (3)	0.63213 (12)	0.0406 (6)	
C8	0.3061 (3)	0.3630 (4)	0.69154 (13)	0.0483 (6)	
H8	0.2866	0.3871	0.7414	0.058*	
C9	0.3967 (3)	0.2032 (4)	0.67709 (14)	0.0567 (7)	
H9	0.4394	0.1194	0.7175	0.068*	
C10	0.4262 (3)	0.1639 (4)	0.60287 (15)	0.0572 (7)	
H10	0.4876	0.0543	0.5934	0.069*	
C11	0.3640 (3)	0.2882 (3)	0.54405 (13)	0.0481 (6)	
H11	0.3841	0.2615	0.4944	0.058*	

### Atomic displacement parameters (Å^2^)

**Table:**

	*U*^11^	*U*^22^	*U*^33^	*U*^12^	*U*^13^	*U*^23^
N1	0.0615 (14)	0.0559 (13)	0.0319 (11)	0.0024 (11)	0.0107 (9)	0.0008 (9)
O1	0.0808 (13)	0.0632 (11)	0.0295 (9)	0.0095 (10)	0.0140 (8)	−0.0030 (8)
O2	0.0956 (15)	0.0587 (12)	0.0470 (11)	0.0183 (11)	0.0150 (10)	−0.0060 (9)
O3	0.0894 (14)	0.0585 (11)	0.0271 (8)	0.0131 (10)	0.0142 (8)	−0.0018 (7)
C1	0.0577 (17)	0.0501 (15)	0.0351 (13)	−0.0025 (13)	0.0100 (10)	−0.0013 (11)
C2	0.0395 (14)	0.0437 (13)	0.0291 (11)	−0.0061 (10)	0.0074 (9)	−0.0026 (10)
C3	0.0402 (14)	0.0482 (14)	0.0331 (12)	−0.0072 (11)	0.0072 (10)	−0.0003 (10)
C4	0.0650 (18)	0.0524 (15)	0.0454 (14)	0.0074 (14)	0.0109 (12)	0.0061 (12)
C5	0.0410 (14)	0.0423 (13)	0.0343 (12)	−0.0040 (11)	0.0101 (10)	−0.0030 (10)
C6	0.0385 (13)	0.0441 (13)	0.0304 (11)	−0.0061 (11)	0.0056 (9)	0.0018 (10)
C7	0.0434 (14)	0.0433 (13)	0.0349 (12)	−0.0046 (11)	0.0060 (9)	−0.0011 (10)
C8	0.0493 (16)	0.0619 (16)	0.0338 (13)	−0.0047 (13)	0.0078 (11)	0.0089 (12)
C9	0.0494 (17)	0.0666 (18)	0.0532 (16)	0.0012 (14)	0.0067 (12)	0.0243 (13)
C10	0.0520 (17)	0.0590 (16)	0.0622 (17)	0.0088 (14)	0.0144 (13)	0.0145 (14)
C11	0.0470 (15)	0.0562 (15)	0.0438 (14)	0.0009 (13)	0.0151 (11)	−0.0003 (12)

### Geometric parameters (Å, °)

**Table:**

N1—C3	1.283 (3)	C5—C6	1.434 (3)
N1—O1	1.435 (2)	C5—H5	0.9300
O1—C1	1.366 (3)	C6—C11	1.392 (3)
O2—C1	1.201 (3)	C6—C7	1.413 (3)
O3—C7	1.346 (3)	C7—C8	1.382 (3)
O3—H3	0.8200	C8—C9	1.363 (3)
C1—C2	1.467 (3)	C8—H8	0.9300
C2—C5	1.359 (3)	C9—C10	1.389 (4)
C2—C3	1.434 (3)	C9—H9	0.9300
C3—C4	1.479 (3)	C10—C11	1.364 (3)
C4—H4A	0.9600	C10—H10	0.9300
C4—H4B	0.9600	C11—H11	0.9300
C4—H4C	0.9600		
			
C3—N1—O1	107.22 (17)	C6—C5—H5	113.4
C1—O1—N1	109.63 (16)	C11—C6—C7	117.43 (19)
C7—O3—H3	109.5	C11—C6—C5	125.0 (2)
O2—C1—O1	119.1 (2)	C7—C6—C5	117.5 (2)
O2—C1—C2	134.3 (2)	O3—C7—C8	121.2 (2)
O1—C1—C2	106.7 (2)	O3—C7—C6	118.35 (19)
C5—C2—C3	124.1 (2)	C8—C7—C6	120.4 (2)
C5—C2—C1	132.6 (2)	C9—C8—C7	120.1 (2)
C3—C2—C1	103.26 (18)	C9—C8—H8	120.0
N1—C3—C2	113.2 (2)	C7—C8—H8	120.0
N1—C3—C4	119.3 (2)	C8—C9—C10	120.9 (2)
C2—C3—C4	127.5 (2)	C8—C9—H9	119.6
C3—C4—H4A	109.5	C10—C9—H9	119.6
C3—C4—H4B	109.5	C11—C10—C9	119.2 (3)
H4A—C4—H4B	109.5	C11—C10—H10	120.4
C3—C4—H4C	109.5	C9—C10—H10	120.4
H4A—C4—H4C	109.5	C10—C11—C6	122.0 (2)
H4B—C4—H4C	109.5	C10—C11—H11	119.0
C2—C5—C6	133.2 (2)	C6—C11—H11	119.0
C2—C5—H5	113.4		
			
C3—N1—O1—C1	−1.5 (3)	C1—C2—C5—C6	2.1 (4)
N1—O1—C1—O2	−179.2 (2)	C2—C5—C6—C11	4.8 (4)
N1—O1—C1—C2	1.5 (2)	C2—C5—C6—C7	−176.4 (2)
O2—C1—C2—C5	0.0 (5)	C11—C6—C7—O3	−178.9 (2)
O1—C1—C2—C5	179.1 (2)	C5—C6—C7—O3	2.2 (3)
O2—C1—C2—C3	179.9 (3)	C11—C6—C7—C8	−0.1 (3)
O1—C1—C2—C3	−1.0 (2)	C5—C6—C7—C8	−179.0 (2)
O1—N1—C3—C2	0.9 (3)	O3—C7—C8—C9	179.2 (2)
O1—N1—C3—C4	−179.32 (19)	C6—C7—C8—C9	0.3 (4)
C5—C2—C3—N1	180.0 (2)	C7—C8—C9—C10	−0.5 (4)
C1—C2—C3—N1	0.0 (3)	C8—C9—C10—C11	0.3 (4)
C5—C2—C3—C4	0.2 (4)	C9—C10—C11—C6	−0.1 (4)
C1—C2—C3—C4	−179.7 (2)	C7—C6—C11—C10	−0.1 (3)
C3—C2—C5—C6	−177.8 (2)	C5—C6—C11—C10	178.7 (2)

### Hydrogen-bond geometry (Å, °)

**Table:**

*D*—H···*A*	*D*—H	H···*A*	*D*···*A*	*D*—H···*A*
O3—H3···N1^i^	0.82	2.07	2.852 (2)	159

Symmetry codes: (i) *x*, −*y*+3/2, *z*+1/2.
